# Parental upheaval experienced in childhood and its effect on pain-related cognitions across the lifespan: an exploratory investigation

**DOI:** 10.3389/fpain.2026.1760192

**Published:** 2026-04-23

**Authors:** Caitlin Curry, Guillermo Ceniza-Bordallo, Emma Costello, Dirichi Ezeh, Margaret Moreland, Christine B. Sieberg

**Affiliations:** 1 Center for Health Outcomes and Interdisciplinary Research, Department of Psychiatry, Massachusetts General Hospital, Boston, MA, United States; 2 Department of Psychiatry, Harvard Medical School, Boston, MA, United States; 3 Division of Adolescent and Young Adult Medicine, Department of Pediatrics, Boston Children’s Hospital, Boston, MA, United States

**Keywords:** divorce, lifespan, pain, post-surgical, upheaval

## Abstract

**Background:**

Chronic post-surgical pain (CPSP), pain lasting at least three months past the expected recovery time of a surgery, affects up to 30% of post-surgical populations. It is multifaceted and influenced by biological and psychological factors. One such factor is exposure to adverse childhood experiences (ACEs), with previous research indicating that exposure to a greater number of ACEs increases the risk of chronic pain development. One of the most frequently experienced types of ACEs is parental upheaval, including divorce and separation, yet its impact on pain-related cognitions and experiences remains understudied.

**Methods:**

65 individuals with CPSP were included in this cross-sectional analysis. Individuals provided self-reported data on their childhood trauma history, as well as measures related to pain perception. Individuals were sorted into two groups based on parental upheaval status: upheaval (*n* = 18) and non-upheaval (*n* = 47).

**Results:**

Participants in the upheaval group demonstrated a more globally connected pattern with pain-related cognitions, anxiety, and somatic arousal contributing to pain interference, while in the non-upheaval group, pain catastrophizing was the central factor influencing interference. Across the sample, pain catastrophizing not parental upheaval status was the most significant variable related to CPSP development. Finally, age and perceived trauma intensity (of the parental upheaval) were positively correlated.

**Conclusion:**

CPSP is a complex condition and warrants biopsychosocial research to elucidate the mechanisms contributing to its onset and maintenance. This study indicates that parental upheaval, one of the more common traumatic events that can happen in childhood, may play an indirect role in pain experiences, potentially increasing vulnerability to maladaptive pain-related cognitions. These findings highlight the importance of considering the influence of ACEs and how they can impact development and subsequently lead to pain-related outcomes across the lifespan.

## 1 Introduction

Chronic post-surgical pain (CPSP) is pain that persists at least 3 months after surgery, with prevalence rates varying widely across surgical procedures (1). Median incidence across surgical types suggests that 10%–40% of individuals experience CPSP at 6–12 months postoperatively (1). Further, CPSP can have a profound impact on patients and their families; for instance, individuals with CPSP are at increased risk of problematic opioid use as they attempt to manage persistent pain with limited effective treatment options (1). Beyond reduced quality of life, CPSP represents a major public health concern that imposes a substantial financial burden on patients and the broader healthcare system through increased healthcare utilization, medication costs, and lost productivity (1).

Because of the high prevalence and variance in presentation and impact, attention has been turned to potential risk factors. Biological risk factors include the type of surgery, as well as pre- and post-operative pain intensity levels (2). Psychological risk factors such as heightened fear, anxiety, and pain catastrophizing also contribute to the development and persistence of CPSP (2). Additionally, exposure to social-environmental influences, including traumatic events in childhood, is also a potential risk factor for the development and maintenance of chronic pain, but their role is less clear (3).

Adverse childhood experiences (ACEs) are potentially traumatic events that occur before the age of 17, collectively referring to abuse, neglect, and household dysfunction (4), and can include the loss of a family member, criminal behavior of a family member, natural disasters, and economic burden (5). With approximately 64% of adults in the U.S. reporting having experienced at least one ACE in their life (6), it is crucial to understand how ACEs can impact pain experiences.

One of the most common types of ACEs is household dysfunction, specifically parental upheaval, such as a divorce or separation (4), with 28% of individuals in the U.S. endorsing this experience (6). Parental upheaval can alter the dynamics of the household, changing a child's environment and introducing instability at a time when their development thrives on reliability and familiarity (7). As a result, adults who experienced parental divorce or separation as children are at an increased risk of psychiatric disorders, problematic substance use, and suicidal ideation and attempts (8).

The associations between parental upheaval and physical health outcomes, including pain, remain unclear. Whereas some studies have found no significant associations between upheaval and chronic pain (9), others suggest that exposure to parental upheaval increases the risk of developing chronic pain (10, 11). Specifically, one study involving more than 6,000 adults ages 35–75 years found that individuals with a history of parental divorce or separation had a higher risk of developing multisite chronic pain and a higher risk of developing neuropathic pain characteristics compared to those without a history of parental divorce or separation (10). The potential impact of parental upheaval on CPSP maintenance and interference remains unknown. This represents a critical gap in the literature, as early exposure to parental conflict, separation, or divorce is known to heighten stress reactivity and dysregulate physiological systems involved in pain modulation (7, 8).

Chronic stress has been consistently identified as a significant predictor of CPSP development, contributing to sensitization of nociceptive pathways, impaired endogenous pain inhibition, and maladaptive emotional regulation (12). Thus, parental upheaval may constitute a form of early-life stress that increases vulnerability to persistent postsurgical pain, yet this potential pathway has not been systematically investigated.

The aims of this exploratory study were to: (1) examine whether participants with CPSP differ in key clinical variables—pain intensity, pain interference, pain catastrophizing, fear/somatic arousal, and anxiety—based on parental upheaval status (upheaval vs. no upheaval), (2) explore the strength and pattern of associations between pain-related outcomes (pain intensity and pain interference) and psychological variables (pain catastrophizing, fear/somatic arousal, and anxiety) in participants with CPSP, stratified by parental upheaval status, (3) investigate whether higher levels of perceived trauma intensity related to parental upheaval are associated with worse clinical outcomes within the upheaval group, and (4) identify potential predictors (parental upheaval status and psychological factors) of pain interference in patients with CPSP.

We hypothesize that participants with CPSP who have experienced parental upheaval will report higher levels of pain intensity, pain interference, pain catastrophizing, fear/somatic arousal, and anxiety, compared to those who have not experienced parental upheaval. Furthermore, we hypothesize that while psychological variables—specifically pain catastrophizing, fear/somatic arousal, and anxiety—will show significant correlations with pain-related outcomes (pain intensity and pain interference) in both groups (upheaval and non-upheaval), that the pattern and strength of these associations may differ between groups, with those who experienced parental upheaval demonstrating worse clinical outcomes (e.g., higher pain interference and anxiety). We anticipate that in the upheaval group, higher ratings of perceived trauma will be related to worse clinical outcomes as well. Finally, we hypothesize that, beyond demographic and clinical factors (age, gender, pain intensity), parental upheaval status and psychological factors—specifically higher anxiety, and higher catastrophizing—will significantly predict higher levels of pain interference in participants with CPSP.

## 2 Method

### 2.1 Study type

This exploratory cross-sectional study was approved by the Institutional Review Boards at two major metropolitan hospitals in the Northeast United States and adhered to the principles of the Declaration of Helsinki. It was conducted following the Strengthening the Reporting of Observational Studies in Epidemiology (STROBE) guidelines for cross-sectional studies (13).

### 2.2 Recruitment process

This data came from two larger studies examining the biopsychosocial factors predicting the development and maintenance of CPSP. Participants included in this sample had undergone surgery at least three months prior to their study visit and were experiencing surgery-related pain that persisted or increased post-operatively for at least three months following surgery, meeting criteria for CPSP. To be included in this sample, participants had to be 18 years of age or older and had to be on a stable dose of any medications they were taking for at least 30 days prior to their visit.

Exclusion criteria included a self-reported history of severe psychological or neurological disorders (e.g., psychosis, schizophrenia), recent or current illicit drug use, current use of antipsychotic medication, or a traumatic brain injury within the past 12 months. Individuals were also excluded if they had severe cognitive impairments or were not sufficiently fluent in English to complete study procedures.

### 2.3 Procedure

Participants were recruited from two large academic hospitals in the northeastern United States, as well as from flyers posted in the hospitals and community, a hospital-based recruitment platform, and social media advertisements.

Upon seeing recruitment materials, participants expressed interest via phone, email, or recruitment platform, and left their contact information. Participants were then contacted by a clinical research coordinator by electronic message and phone, before completing a phone screening survey to determine their eligibility to participate. Those who were deemed eligible following the phone screening were emailed more information, and a time was arranged for their visit.

Informed consent was obtained at the beginning of the participant's study visit. Data was collected at in-person study visits. Information about medical history and all self-report psychosocial data was collected using Redcap software (14, 15).

#### 2.3.1 Psychosocial measures

Using the NIH Toolkit's *Fear/Somatic Arousal* questionnaire (16), symptoms of anxiety associated with autonomic arousal were assessed. This six-item, five-point Likert-style questionnaire asks people to rate how frequently in the past seven days they have experienced certain sensations such as “I felt dizzy or lightheaded”, with answers ranging from “not at all” to “extremely”. Higher scores on this measure indicate higher levels of somatic arousal. Scores one standard deviation (SD) or higher above the mean (greater than or equal to 60) are considered to be high, and scores one SD or lower below the mean (less than or equal to 40) are considered to be low (16). Cronbach's alpha for this measure in this data set was.75. Previous studies have found this measure to be valid and reliable for participants in pain (16).

Anxiety levels in the past seven days were measured using the *PROMIS Anxiety Short Form* (PROMIS Anxiety-SF) (17). This self-assessment scale consists of eight items with five-option Likert-type responses ranging from “never” to “always”. PROMIS Anxiety-SF T-scores ranged from 37.1 to 75.4, where higher scores indicated higher levels of anxiety. Cronbach's alpha for this measure in this data set was.99. Previous studies have found this measure to be valid, including in populations with various chronic conditions, including chronic pain (18).

The *PROMIS Pain Interference Short Form* (PROMIS-PI-SF) (19) was used to measure the degree to which an individual felt that their pain was impacting their day-to-day life, in elements such as social life and work life (19). This is a self-rated, eight-item, five-point Likert-style questionnaire. It asks individuals to endorse the extent to which the statements resonate with their experiences in the past seven days. PROMIS-PI-SF T-scores ranged from 40.7 to 77.0, where higher scores indicated higher levels of pain interference. Cronbach's alpha for this measure in this data set was.95. This measure is valid in several populations, including chronic pain populations (19).

The *Pain Catastrophizing Scale* (PCS) (20) was used to assess negative thoughts and worries associated with pain. This is a 13-item self-assessment questionnaire with a five-point Likert response style. Response options range from “not at all” to “all the time”. This scale is measured in total score as well as subscores, which collectively examine the extent to which individuals ruminate on, magnify, and feel helpless in their pain. Total PCS scores ranged from 0 to 44, with higher scores indicating higher pain-related worries and negative thinking. Cronbach's alpha for this measure in this data set was .92. This measure has been found to be valid in both non-clinical and clinical samples (20).

Experiences of childhood trauma were measured using the *Childhood Traumatic Events Scale and Recent Traumatic Events Scale* (CTES/RTES) (21). For this analysis, we specifically looked at whether or not individuals endorsed having experienced parental upheaval. The question asked, “prior to the age of 17, was there a major upheaval between your parents (such as divorce, separation)?”, and asked them to rate, 1(not at all traumatic)-7 (extremely traumatic) how traumatic the upheaval was.

#### 2.3.2 CPSP determination

CPSP status was determined based on participants’ self-report responses to a standardized questionnaire about medical history. Participants answered a series of yes or no questions followed by a free-response question to elaborate on their answer. The questions included: “Did you have any have post-surgical pain longer than the expected recovery time?” followed by a free response question, “Please explain (duration of pain, nature of pain, etc.)”, “Did any of the surgeries result in complications?” followed by “Please explain”, and “Are you experiencing pain at the site of your surgery today?” followed by “Please rate your pain (scale of 0–10) today and describe the location of the pain”. Responses were reviewed by multiple research team members, and individuals who reported experiencing pain related to a surgery that occurred at least three months prior to the time of their study visit were considered to have CPSP.

### 2.4 Statistical analysis

All statistical analyses were conducted using IBM SPSS Statistics version 29.0.2 (SPSS, Inc., Chicago, IL, USA) (22), with the level of statistical significance set at *p* < .05 (two-tailed) for all tests. Descriptive statistics were computed for demographic, clinical, and psychological variables. For continuous variables (e.g., age, pain intensity, anxiety, catastrophizing, pain interference, fear and somatic arousal), means, standard deviations, medians, and interquartile ranges were reported depending on the distribution of the data. For categorical variables (e.g., gender, parental upheaval status), absolute and relative frequencies (percentages) were calculated.

To address the first aim, participants were divided into two groups based on parental upheaval status (upheaval vs. no upheaval). Group differences were examined across all relevant demographic, clinical, and psychological variables. Independent samples t-tests were used for continuous variables with normal distributions; Mann–Whitney *U*-tests were used when normality was not assumed. Chi-square tests (or Fisher's exact tests when appropriate) were used for categorical variables. Group comparisons included: pain intensity, pain interference, anxiety, catastrophizing (and subscales), fear and somatic arousal, age and gender.

To address the second aim, Pearson's correlation coefficients (or Spearman's rho in case of non-normal distributions) were computed separately for participants with CPSP, stratified by parental upheaval status (upheaval vs. no upheaval). Correlation strength was interpreted as follows: coefficients of .10–.29 were considered weak, *.*30–.49 moderate, and .50 or greater were considered strong (23).

To address the final aim, we conducted multiple linear regression analyses including four blocks. For each planned regression model, we followed the same strategy. We first introduced participants’ demographic characteristics (i.e., age, gender) in a model as control variables. We then introduced pain intensity (Model 2), catastrophizing, fear and somatic arousal, anxiety (Model 3) and upheaval status (Model 4). The models were evaluated by reporting standardized regression coefficients (β) with 95% confidence intervals. Comparing adjusted *R*^2^ values to assess the additional explained variance contributed by psychological variables. Multicollinearity was assessed through Variance Inflation Factors (VIF), ensuring all VIF values were below 5 (24). Regression assumptions were checked via residual diagnostics (linearity, normality, homoscedasticity).

### 2.5 Sample size

Sample size considerations for group comparisons and correlations (Aims 1 and 2): Given the available sample size (*N* = 65), a sensitivity analysis was conducted to determine the minimum effect sizes detectable with adequate power. For the group comparisons (Aim 1), assuming a 2:3 group ratio (e.g., *n* = 25 vs. 40), the study was sufficiently powered (1 − β = .80) to detect large between-group differences (Cohen's d ≥ *.*80) at α = *.*05*.* For the stratified correlational analyses (Aim 2), the minimum detectable correlation within each subgroup (*n* ≈ 30) was *r* ≥ *.*50, corresponding to a strong effect. Therefore, while the study may be underpowered to detect small or moderate effects, it remains suitable for identifying large and clinically meaningful differences and associations.

Sample size considerations for hierarchical regression (Aim 3): An *a priori* power analysis was conducted for the hierarchical linear regression examining whether psychological variables (anxiety, catastrophizing, fear and somatic arousal) explained additional variance in pain interference beyond demographic and clinical predictors (age, gender, pain intensity, and parental upheaval status). Given prior literature highlighting the role of psychological factors in pain-related outcomes, we anticipate a medium effect size (*f²* = 0.15) for the second block of predictors (anxiety, catastrophizing, and fear and somatic arousal). This is consistent with Cohen's conventional guidelines for effect size in multiple regression. With an α level of *.*05, power of .80, and a total of 7 predictors (4 in the first block, 3 in the second), the minimum required sample size was estimated at 71 participants. Given the available sample size (*n* = 65), the analysis is slightly underpowered, and results should be interpreted as exploratory.

## 3 Results

### 3.1 Participants

277 potential participants were screened to determine eligibility to participate in the larger studies. Of those screened, 205 were deemed eligible to participate, and 146 of them were enrolled in the study. Of those who enrolled, 65 individuals endorsed having CPSP and were included in this sample. All participants included in this study completed the surveys and provided full data on the variables of interest. No cases were excluded due to missing data, and no outliers were identified.

### 3.2 Sample characteristics and differences across upheaval and non-upheaval groups

Participants’ demographic and clinical characteristics are presented in Table 1, and surgical characteristics can be found in Table 2. The majority of surgeries related to CPSP were orthopedic (e.g., knee, spine), while other surgeries (31%) included procedure types such as cosmetic, gender affirmation, and orofacial. Most participants were female (80%) and identified as White, non-Hispanic (83.1%). There were no significant differences in gender, age, ethnicity, or race between the upheaval and non-upheaval groups (see Table 3). Regarding upheaval status, 27.7% (*n* = 18) reported experiencing a recent major life upheaval (e.g., divorce), whereas 72.3% (*n* = 47) did not.

**Table 1:** Demographics and clinical characteristics of the participants included in this study (*n* = 65).

Variable	Mean (SD)/ % (*n*)	Median (IQR)	Range (min-max)
Age (years)*		29 (30)	18–80
Gender			
Male	20% (13)	–	–
Female	80% (52)	–	–
Ethnicity			
Non-Spanish	83.1% (54)	–	–
Spanish	15.5% (10)	–	–
Race	–	–	–
White or Caucasian	73.84% (48)		
Black or African American	9.23% (6)	–	–
Asian or Pacific Islander	6.15% (4)	–	–
Multiracial or Biracial	6.15% (4)	–	–
Unknown	3.07% (2)	–	–
Middle Eastern	1.54% (1)	–	–
Upheaval status			
Upheaval	27.7% (18)	–	–
Non-upheaval	72.3% (47)	–	–
Intensity of trauma (divorce)*^,^^	5.00 (1.68)	5 (3)	2–7
Pain intensity*		3 (5.50)	0–10
Pain catastrophizing*		14 (12)	0–44
Magnification	–	2 (2)	0–9
Rumination	–	5 (5)	0–16
Helplessness	–	6 (7)	0–19
Pain interference	55.97 (7.05)	–	40.7–77.0
Fear and somatic arousal*	–	52.5 (12.2)	37.9–87.9
Anxiety*	–	54.3 (12.6)	37.1–63.5

* Non-normal distribution.

^ *n*:18 participants.

**Table 2:** Surgical characteristics.

Most recent surgery to study visit (%)	Time since surgery	*n* = 65
	>1 year	24
	1–2 years	19
	2–3 years	6
	3–4 years	1
	4 + years	13
Type of surgery related to post-surgical pain (%)		
	Orthopedic	46 (69)
	Other	21 (31)
Pain Experiences		*n* = 65
Baseline Pain Score		3.41 ± 2.68 (0–10)
Pain at surgical site (%)		100
Pain lasting longer than 3 months (%)		100
Pain Level in Past Week		4.46 ± 2.05 (1–8)

**Table 3:** Comparisons between upheaval and non-upheaval groups across demographic and clinical variables.

Variable	Not Upheaval (*n* = 47) Median [IQR]/Mean SD/%(n)	Upheaval (*n* = 18) Median [IQR]/Mean SD/ % (n)	*p*-value	Effect size
Age	27.5 [31]	40.5 [30]	.202^	–
Gender-Male	17% [8]	27% [5]	.245*^σ^*	–
Gender-Female	83% [39]	72.2% [13]	.357^σ^	–
Pain intensity	3 [5]	3 [6]	.344^	–
Pain catastrophizing	14 [12]	18 [19]	.159^	–
Magnification	2 [3]	3 [3]	.087^	–
Rumination	5 [4]	6 [7]	.302^	–
Helplessness	6 [7]	7.5 [11]	.216^	–
Pain interference	54.76 [6.37]	59.13 [7.91]	.175*	–
Fear and somatic arousal	**52.5 [9.6]**	**57.15 [13.4]**	**.** **038^**	**0**.**258**^
Anxiety	52.1 [13.5]	57.90 [10.6]	.214^	–

Values are presented as median [IQR] for non-normally distributed variables and mean (SD) for normally distributed variables. *p*-values are based on Mann–Whitney *U*-test (^), *t*-test (*), or Fisher's exact test (*σ*), as appropriate. Effect sizes (Rosenthal's r) are reported for significant results. Bold values indicate statistically significant group differences (*p* < .05).

Mild pain intensity was found (*median* = 3) across the whole sample, lacking statistically significant differences between upheaval and non-upheaval groups (*p* > .05). Pain catastrophizing and anxiety were mild in the whole sample (*median* = 14, 54.3, respectively). There were no significant differences between groups on pain catastrophizing (14 vs. 18, *Mann–Whitney U test p* = .159) or anxiety (52.1 vs. 57.9, *Mann–Whitney U-test p* = .214). Additionally, no significant differences in pain catastrophizing subscale scores (magnification, rumination, and helplessness) were observed (*Mann–Whitney U-test p* = .087;.302;.216, respectively). Across the whole sample, pain interference was mild (55.97), and differences between upheaval and non-upheaval groups were not significant (54.84 vs. 59.13 respectively, *p* = .175).

Fear and somatic arousal scores were mild (52.50), and there were significant differences observed between the upheaval and non-upheaval groups with a moderate effect size (*Cohen d* = 0.258), indicating that individuals with a history of parental upheaval in childhood experience higher fear and somatic arousal than those who did not experience a parental upheaval.

### 3.3 Correlations among clinical variables in whole sample

Correlations among clinical variables for the total sample are shown in Table 4. The results showed age was inversely correlated with helplessness (*rho =* −.253) and anxiety (*rho* = −.357), with younger participants tending to experience greater feelings of helplessness and higher anxiety levels in relation to their pain. Pain intensity was directly correlated with helplessness (*rho* = .252) and pain interference (*rho* = .264), suggesting that participants reporting higher pain intensity also perceive their pain as more impactful and feel less capable of managing it.

**Table 4:** Correlations among variables for the whole sample (*n* = 65).

Variables	1	2	3	4	5	6	7	8	9
1. Age	—								
2. Pain intensity	.091	—							
3. Pain catastrophizing	–.211	.193	—						
4. Magnification	–.231	.019	.803***	—					
5. Rumination	–.017	.153	**.** **898** ***	**.** **656** ***	—				
6. Helplessness	–.253*	.252*	.905***	**.** **610** ***	**.** **703** ***	—			
7. Pain interference	.055	.264*	**.** **589** **	**.** **366** **	**.** **479** ***	**.** **652** ***	—		
8. Fear and somatic arousal	–.199	.093	**.** **392** **	**.** **352** **	**.** **266** *	**.** **368** **	**.332** **	—	
9. Anxiety	–.357**	−.063	**.** **576** **	**.** **603** ***	**.** **437** ***	**.** **477** ***	.152	**.392** **	—

Spearman's rho for all variables; Bold values indicate statistically significant correlation.

* *p* < .05.

** *p* < .01.

*** *p* < .001.

5w?>Regarding cognitive pain processes, higher pain catastrophizing was associated with greater pain interference (*rho* = .589), fear and somatic arousal (*rho =* .392), and anxiety (*rho =* .576). The subcomponents of catastrophizing also followed this pattern. Helplessness correlated strongly with rumination (*rho* = .703) and magnification (*rho* = .610) and moderately with pain interference (*rho* = .652,), fear and somatic arousal (*rho* = .368) and anxiety (*rho* = .477). This suggests that participants who feel unable to manage pain tend to ruminate more and experience both cognitive and physiological amplification of their symptoms. Moreover, rumination and magnification also correlated with anxiety (*rho* = .437,.603 respectively), supporting notions that repetitive negative thinking about pain is closely linked to affective distress.

Clinically, these correlations illustrate a coherent pattern in which maladaptive cognitive responses to pain—such as catastrophizing, helplessness, and rumination—are tightly interwoven with emotional dysregulation (anxiety) and functional impairment (pain interference).

### 3.4 Correlations among clinical variables in non-upheaval group

When examining the correlation patterns separately for the non-upheaval and upheaval groups, distinct relational profiles emerged between cognitive, emotional, and functional pain-related variables.

Correlations among variables in the non-upheaval group can be found in Table 5. In the non-upheaval group (*n* = 47), age was inversely correlated with catastrophizing (*rho* = −.533), helplessness (*rho* = −.582), magnification (*rho* = *–.*476*),* fear and somatic arousal (*rho = –.*319*),* and anxiety (*rho* = −.565), pointing toward age related differences demonstrating improved emotional regulation and more adaptive pain cognitions in older participants. Catastrophizing was also moderately correlated to pain interference (*rho =* .603) and anxiety (*rho =* .555), suggesting that participants who showed higher catastrophic thinking tend to experience both greater disruption in daily functioning and higher emotional distress. Also, the results showed that anxiety was correlated with fear and somatic arousal (*rho* = .340), indicating that participants who experience higher anxiety also tend to show greater emotional and physiological reactivity to pain. This suggests that anxious individuals may display heightened bodily tension and vigilance toward pain-related sensations, reflecting a stronger activation of the pain-related fear response system.

**Table 5:** Correlations among variables for the non-upheaval group (*n* = 47).

Variables	1	2	3	4	5	6	7	8	9
1. Age	—								
2. Pain intensity	–.013	—							
3. Pain catastrophizing	**–** **.** **533** **	.176	—						
4. Magnification	**–** **.** **476** **	–.070	**.** **777** **	—					
5. Rumination	–.203	.119	**.** **850** **	**.** **579** **	—				
6. Helplessness	**–** **.** **582** **	.126	**.** **889** **	**.** **567** **	**.** **621** **	—			
7. Pain interference	–.137	.055	**.** **603** **	**.** **397** **	**.** **502** **	**.** **591** **	—		
8. Fear and somatic arousal	**–** **.** **319** *	.025	.280	.206	.138	.240	.250	—	
9. Anxiety	**–** **.** **565** **	–.117	**.** **555** **	**.** **487** **	**.** **360** *	**.** **489** **	.149	**.** **340** *	—

Spearman's rho for all variables; Bold values indicate statistically significant correlation.

* *p* < .05.

** *p* < .01).

### 3.5 Correlations among variables in upheaval group

In contrast, the upheaval group (*n* = 18) displayed a more globally interconnected pattern, with stronger associations extending beyond cognitive dimensions. Correlations among variables in the upheaval group can be found in Table 6. Pain intensity correlated positively with age (*rho* = .570), pain interference (*rho* = .632). Age is correlated with intensity of the trauma (*rho* = .581), indicating that older participants in this subgroup reported higher pain levels and greater perceived impact of traumatic experiences. Catastrophizing and its components again formed a tightly linked cluster (magnification: *rho* = .814, rumination: *rho* = .946, helplessness: *rho* = .940), but in this group, these cognitive variables were more strongly coupled with affective symptoms such as fear and somatic arousal (*rho* = .701–.707) and anxiety (*rho* = .643–.897). Additionally, pain interference showed a strong correlation with helplessness (*rho* = .801), emphasizing the functional toll of maladaptive pain cognitions in participants exposed to upheaval.

**Table 6:** Correlations among variables for the upheaval group (*n* = 18).

Variables	1	2	3	4	5	6	7	8	9	10
1. Age	—									
2. Pain intensity	**.** **570** *	—								
3. Pain catastrophizing	.452	.314	—							
4. Magnification	.240	.043	**.** **814** **	—						
5. Rumination	.363	.197	**.** **946** **	**.** **781** **	—					
6. Helplessness	**.** **506** *	.438	**.** **940** **	**.** **666** **	**.** **818** **	—				
7. Pain interference	**.** **483** *	**.** **632** **	**.** **609** **	.249	.446	**.** **801** **	—			
8. Fear and somatic arousal	.042	.214	**.** **701** **	**.** **633** **	**.** **556** *	**.** **707** **	**.** **476** *	—		
9. Anxiety	.140	–.067	**.** **643** **	**.** **897** **	**.** **627** **	.441	.077	.475*	—	
10. Intensity of trauma	**.** **581** *	.215	.464	.161	.449	**.** **500** *	.360	.218	–.031	—

Spearman's rho for all variables; Bold values indicate statistically significant correlation.

* *p* < .05.

** *p* < .01).

Comparatively, while both groups demonstrated the expected cohesion among catastrophizing subscales, the upheaval group showed a more generalized pattern of emotional interdependence, with stronger links between pain-related cognitions, anxiety, physiological arousal, and trauma intensity. Conversely, the non-upheaval group exhibited a more compartmentalized pattern, where catastrophizing remained central but with clearer age-related associations (younger age linked to greater helplessness and anxiety). This suggests that exposure to upheaval may blur the boundaries between cognitive, emotional, and functional domains of pain processing, creating more integrated and potentially maladaptive pain-related cognitions.

### 3.6 Regression modeling to predict pain interference

Results of the regression analysis are presented in Table 7. In Model 1, age and gender were entered as predictors and were not significantly associated with pain interference. In Model 2, age, gender, and pain intensity were not significantly associated with pain interference. Model 2 did not significantly improve model fit (Δ*F* = 0.157; *p* = .693). In Model 3, pain catastrophizing and fear and somatic arousal were added, and both pain catastrophizing [*β* = .629, 95% CI (0.269, 0.644), *p* < .001] and fear and somatic arousal [*β* = .254, 95% CI (0.010, 0.341), *p* < .01] emerged as significant predictors. This model significantly increased the explained variance (Δ*F* = 14.35 *p* < .001), indicating that the inclusion of cognitive-emotional factors accounted for an additional 41.4% of the variance in pain interference (Δ*R*^2^ = .414). Contrary to our hypothesis, in Model 4, the upheaval variable was not significantly associated with pain interference, and Model 4 did not result in a significant change in explained variance (Δ*F* = 1.394, *p* = .243). On the other side, pain catastrophizing remained the strongest and most consistent predictor [*β* = .611, 95% CI (0.255, 0.632), *p* < .001].

**Table 7:** Linear hierarchical regression models predicting pain interference.

Predictor	Model 1 β (95% CI)	Model 2 β (95% CI)	Model 3 β (95% CI)	Model 4 β (95% CI)
Age	0.163 (−0.048; 0.184)	0.157 (−0.520; 0.183)	0.124 (−0.049; 0.153)	0.099 (−0.061; 0.143)
Gender (male ref.)	0.184 (−1.640; 8.080)	0.175 (–1.908; 8.027)	−0.024 (−4.428; 3.578)	−0.011 (−4.197; 3.826)
Pain intensity		0.051 (–0.527; 0.788)	0.038 (−0.425; 0.621)	0.070 (−0.361;0.716)
Catastrophizing			**0.629 (0.269; 0.644)** **	**0.611 (0.255; 0.632)** **
Fear and somatic arousal			**0.254 (0.010; 0.341)** *	0.224 (−0.014; 0.323)
Anxiety			−0.250 (−0.461; 0.015)	−0.258 (−0.467; 0.007)
Upheaval				0.130 (−1.412; 5.466)
*R* ^2^	.035	.037	.452	.465
Δ*R*²	–	.003	.414	0.13
ΔF	–	0.157, *p* = .693	14.35, *p* < .001	1.394, *p* = .243

Standardized regression coefficients (β) are shown with 95% confidence intervals. Bold values indicate statistically significant predictors.

* *p* < .05.

** *p* < .01. Gender reference category = male.

## 4 Discussion

The goal of the present exploratory study was to understand the potential impact of a common ACE, parental upheaval, on key affective and cognitive variables in a population of participants with CPSP. The study sought to examine differences between these variables, stratified by whether individuals had experienced an upheaval, and to investigate potential predictive implications of parental upheaval on pain interference in CPSP populations. Of note, the design of this study does not address the development of CPSP onset, but rather the maintenance and pain-related psychological experiences in a population with established CPSP.

The findings of this study suggest that cognitive and emotional factors function as the primary drivers of pain interference. While the initial hypotheses of the study suggested that parental upheaval status would be associated with pain interference and psychological variables, the findings support pain catastrophizing to be the primary driver of pain interference. In this study of individuals with CPSP, pain catastrophizing was found to be the primary predictor of pain interference across the entire sample. These findings align with previous research that has found pain catastrophizing related to pain interference in several clinical samples, including oncological settings (25) and in individuals who identify as disabled (26). In surgical contexts, pain catastrophizing is a known predictor of post-operative outcomes, including health-care utilization and opioid usage (27), in addition to pain-related measures, such as pain intensity and interference (28, 29).

When exploring differences between groups, the upheaval participants showed a more globally interconnected pattern across cognitive, emotional, and functional domains, with stronger associations between pain-related cognitions, anxiety, somatic arousal, and trauma intensity. This contrasted with the non-upheaval group's characteristics, which emphasized catastrophizing as the central feature. Interestingly, in the non-upheaval group, despite being the central feature, age-related differences in catastrophizing and anxiety scores were observed, with older age associated with decreased anxiety and feelings of helplessness. These findings converse with previous research that found age to be a significant moderator in the predictive association between pain catastrophizing and pain interference (30). In this study, age did not emerge as an associated factor in the upheaval group. These findings posit that in CPSP populations who have experienced a parental upheaval, there could be a more integrated and potentially maladaptive psychophysiological mechanisms that may contribute to pain experiences. While the non-upheaval group demonstrated a less integrated profile, wherein age was significantly inversely correlated with catastrophizing and anxiety scores, the profile of the upheaval group does not seem to be impacted by age. These findings compliment previous research suggesting that in healthy adults, emotional well-being maintains or improves across the lifespan (31), while divorce, a common form of parental upheaval, can lead individuals towards less mature coping mechanisms, even throughout the lifespan as individuals age (32). While there were observed differences in psychological variables between the upheaval and non-upheaval groups, the regression model showed that parental upheaval status was not a predictor of pain interference, contrary to expectations (controlling for cognitive and emotional variables).

Considering these findings, we hypothesize that while upheaval does not have a direct effect on pain interference, it may be indirectly impactful – potentially serving as a vulnerability that could amplify maladaptive cognitions and emotional dysregulation, or impact development of coping mechanisms, rather than directly influencing pain outcomes (33, 34). While there is a body of research that finds childhood trauma related to an increase in somatic anxiety and a decline in physical health both in youth and adulthood (34–38), this potential nuanced explanation requires further research on mediational pathways that navigate from trauma-related stress to interference. Furthermore, much of the existing research that considers the impact of childhood trauma on health outcomes does not include parental upheaval or divorce as a trauma considered, warranting an expansion of the research to include this event. For this reason, the findings of the present study are both relevant and highly novel, highlighting the importance of incorporating this variable into future investigations on lifespan components and their long-term effects on health and well-being.

As for the positive relationship between age and perceived trauma intensity in the upheaval group, it is important to consider that self-reported recall of trauma intensity is subject to recall bias which could be influenced by age and psychological state (39). However, these findings could be explained by previous findings suggesting that trauma responses and processing can be delayed or late-onset (40), though there is an obvious gap in understanding this phenomenon in older populations (41). It is possible, though, that these findings are more aligned with research suggesting that generational differences in beliefs about divorce are changing, with older generations and individuals who are more religious being more in favor of upholding the institution of marriage and therefore avoiding divorce, while younger and more LGBTQ+ aligned individuals are more in favor of deinstitutionalizing marriage and of divorce or separation (42). Divorce rates in respective age brackets have increased over time (43), and the fact that they may feel more common and that attitudes are shifting could explain why older adults are more likely to perceive their parental upheaval as more traumatic. As for the observed association between trauma intensity and helplessness, it is possible that this relationship is due to similar impact of cognitive-emotional states as opposed to a direct relationship between the two variables (44).

While we did not find a direct impact of upheaval status on pain interference, differing patterns of pain-related cognitions and worries did emerge between groups. We also found that pain-related cognitions, primarily catastrophizing, are the most impactful predictors of pain interference in this CPSP sample. Drawing on these findings, this study suggests that the development of interventions targeting catastrophic thinking and fear responses could be beneficial in reducing pain interference, especially in individuals with exposure to stressful life events in childhood, including parental upheaval. Additionally, the nature of these findings suggests that it is not the presence of a parental upheaval that impacts pain interference, but the pain-related cognitions individuals experience, which may be influenced by an upheaval. Therefore, it is possible that screening for emotional and cognitive vulnerability, or maladaptive pain-related cognitions, may be more clinically relevant than focusing solely on gaining an inventory of life history and traumatic events.

The findings of this study should be considered alongside the limitations, which provide directions for future research. The cross-sectional design of the study was useful in allowing us to gather data from various life stages. However, to clarify causal links and to gain a better understanding of the relationship between parental upheaval, development, and pain-related outcomes, longitudinal research is warranted. Longitudinal research incorporating a prospective design could help to shed light on these variables as they relate to the development of CPSP as opposed to in a group with already established CPSP.

Additionally, this study relied on self-reported data about psychological variables as well as pain intensity and interference. While self-report measures do provide insight into the participants’ experiences, they are subject to shared method variance and response bias, which can be particularly relevant in constructs related to affect and cognition. To account for this, future research could take a more truly biopsychosocial approach by including multimodal data such as biological measures of stress such as salivary or hair cortisol sampling, more objective pain measures such as quantitative sensory testing, and neuroimaging in addition to self-report measures. This could better illustrate a picture of potential differences in biological pathways as they interact with cognitive and affective experiences. Furthermore, a weakness of these findings is the sample composition and generalizability.

This study drew from a smaller sample size, especially in the subgroup reporting a parental upheaval in childhood, which likely reduced the statistical power to detect group differences or moderation effects. Additionally, the sample primarily consisted of white female participants, and while CPSP does predominantly affect females (45), this could limit the generalizability of these findings to broader clinical and cultural contexts.

While the focus of this study was on parental upheaval, as it is one of the more common adverse childhood events that might be experienced, we did not consider other potential childhood or recent traumatic events that could have been experienced. Previous research does indicate that higher cumulative levels of lifetime trauma are associated with more severe somatic symptoms and nervous dysregulation associated with chronic pain (35–38). In addition, previous research finds that parental upheaval is likely to co-occur with other ACEs including financial instability and conflict (46). While the small sample size in this exploratory study did not allow for the assessment of or control for other ACEs, future research should consider the impact of a parental upheaval alongside other potential trauma history that may influence responses.

In a similar vein, future research could consider controlling for variables such as age at the time of the upheaval and time since the upheaval (at data collection), as these variables could potentially impact the observed relationships. Finally, with these findings, we suggest that the experience of a parental upheaval in childhood may be indirectly affecting pain-related outcomes, such as interference by means of maladaptive coping. As such, future studies should consider introducing measures to assess pain-related coping, to elucidate the potential mechanisms of this connection.

## 5 Conclusions

While it is widely accepted that childhood experiences can shape who people become from a psychological and personal perspective, it is also important to understand their influence on how an individual's body interacts with and processes its environment. Previous research finds that exposure to childhood adversity is associated with impacts on the hypothalamic-pituitary-adrenal (HPA) axis. This can alter nociceptive processing, leading to heightened pain sensitivity and poorer overall health outcomes (47, 48), such as increased risk for the development of chronic pain (5, 49–51).

Despite the limitations of this exploratory study, the findings of this study highlight the importance of pain-related cognitions, primarily pain catastrophizing, in the level of interference experienced by individuals with CPSP. While we expected to see a direct effect of upheaval status on interference, these results suggest that parental upheaval experienced in childhood does not directly influence pain interference, but could lead to the development maladaptive pain-related cognitions, not associated with age. We hypothesize that this pathway could thus indirectly affect the degree to which individuals are impacted by their pain. This research, conducted in a cohort of individuals with CPSP, suggests that interventions targeting maladaptive cognitions, such as catastrophizing and fear, could be useful in reducing pain interference, especially in individuals with exposure to parental upheaval in childhood. Such treatments could include modalities such as Cognitive Behavioral Therapy (52), Acceptance and Commitment Therapy (53), and multimodal therapeutic approaches that incorporate physical movement (54). These treatments have been studied in select pain contexts, but further research is warranted in determining how they would interact with a CPSP sample, especially those who have been exposed to ACEs. Overall these findings warrant further longitudinal and multimodal investigation to get an understanding of the biopsychosocial mechanisms contributing to pain-related experiences in individuals who have experienced stress and trauma in childhood.

## Data Availability

The raw data supporting the conclusions of this article will be made available by the authors, without undue reservation. Contact the corresponding author for access.
